# The glutathione transferase of *Nicotiana benthamiana* NbGSTU4 plays a role in regulating the early replication of *Bamboo mosaic virus*

**DOI:** 10.1111/nph.12304

**Published:** 2013-05-23

**Authors:** I-Hsuan Chen, Meng-Hsuen Chiu, Shun-Fang Cheng, Yau-Heiu Hsu, Ching-Hsiu Tsai

**Affiliations:** 1 Graduate Institute of Biotechnology, National Chung Hsing UniversityTaichung, 402, Taiwan; 2 Graduate Institute of Medical Laboratory Science and Biotechnology, China Medical UniversityTaichung, 404, Taiwan

**Keywords:** *Bamboo mosaic virus* (BaMV), glutathione (GSH), glutathione S-transferase (GST), *in vitro* RNA replication, redox, viral RNA replication, virus-induced gene silencing (VIGS)

## Abstract

- *Bamboo mosaic virus* (BaMV) is a single-stranded positive-sense RNA virus. One of the plant glutathione S-transferase (GST) genes, *NbGSTU4*, responds as an upregulated gene in *Nicotiana benthamiana* post BaMV infection.
- In order to identify the role of NbGSTU4 in BaMV infection, the expression of *NbGSTU4* was knocked down using a virus-induced gene silencing technique or was transiently expressed in *N. benthamiana* in BaMV inoculation.
- The results show a significant decrease in BaMV RNA accumulation when the expression level of *NbGSTU4* is reduced; whereas the viral RNA accumulation increases when *NbGSTU4* is transiently expressed. Furthermore, this study identified that the involvement of *NbGSTU4* in viral RNA accumulation occurs by its participation in the viral early replication step. The findings show that the NbGSTU4 protein expressed from *Escherichia coli* can interact with the 3′ untranslated region (UTR) of the BaMV RNA *in vitro* in the presence of glutathione (GSH). The addition of GSH in the *in vitro* replication assay shows an enhancement of minus-strand but not plus-strand RNA synthesis.
- The results suggest that the plant GST protein plays a role in binding viral RNA and delivering GSH to the replication complex to create a reduced condition for BaMV minus-strand RNA synthesis.

## Introduction

Glutathione S-transferases (GSTs) are grouped into a large family of both eukaryotes and prokaryotes and catalyze a variety of reactions (Sheehan *et al*., 2001; Dixon *et al*., 2002; Allocati *et al*., 2009). Conventionally, GSTs catalyze the transfer of a reduced tripeptide glutathione (GSH; glutamyl-cysteinyl-glycine) to various substrates containing a reactive electrophilic center, to form a polar S-glutathionylated product for reducing oxidative stress (Dixon *et al*., 2002). Plant GSTs were first recognized in 1970, and have been proven to play a role in the detoxification occurring in herbicide injuries (Frear & Swanson 1970). These plant GSTs are classified into eight distinct groups: phi, tau, theta, zeta, lambda, DHAR, TCHQD and microsomal (Edwards & Dixon, 2005). As more plant genomes are sequenced, the list of GSTs continues to grow. In *Arabidopsis*, 48 GST-like genes were grouped into 28 tau, 13 phi, 3 theta, 2 zeta and 2 lambda GSTs (Dixon *et al*., 2002); 42 maize GST-like genes were grouped into 12 phi, 28 tau and 2 zeta GSTs; and 25 soybean GST-like genes were grouped into 20 tau, 1 zeta and 4 phi GSTs (McGonigle *et al*., 2000). The members of the phi and tau family are specific to plants and are the most abundant. They are induced by various treatments that induce general oxidative stress, such as osmotic stress, temperature stress and chemical toxins (Mauch & Dudler, 1993; Marrs, 1996). Furthermore, the role of tau GSTs has been characterized in the tolerance to cold temperature and the oxidative stress that occurs when they are overexpressed in tobacco seedlings (Roxas *et al*., 1997). Similar responses were also observed in yeast (Kampranis *et al*., 2000; Kilili *et al*., 2004). These observations suggest that the tau GSTs can play a significant role in protecting plants against oxidative stress. Moreover, three tau GSTs (*NbGSTU1*, *NbGSTU2* and *NbGSTU3*) and one phi GST (*NbGSTF1*) from *Nicotiana benthamiana* plants were examined for their roles in fungal infections (Dean *et al*., 2005). The expression levels of *NbGSTU1* and *NbGSTU3* were found to be upregulated post-infection, although those of *NbGSTU2* and *NbGSTF1* were unaffected. Further analysis revealed that only the *NbGSTU1*-knockdown plant showed more lesions compared to the others when inoculated with *Colletotrichum orbiculare*. These findings suggest that, although a relatively large number of different GSTs are present in plants, only a few are involved in disease development (Dean *et al*., 2005).

*Bamboo mosaic virus* (BaMV) is a single-stranded positive-sense RNA virus. An RNA genome of *c*. 6.4 kb with a 5′-cap and a 3′-poly(A) tail contains five open-reading frames (Lin *et al*., 1992, 1994). ORF1 encodes a 155 kDa replicase for viral RNA replication (Li *et al*., 2001; Huang *et al*., 2004). ORF2 to ORF4 encode movement proteins that participate in intra- and intercellular movement of the virus (Lin *et al*., 2004, 2006). ORF5 encodes the capsid protein for virus encapsidation. Potexvirus coat protein is also involved in virus movement (Cruz *et al*., 1998). The 3′ untranslated region (UTR) of BaMV has been structurally mapped (Cheng & Tsai, 1999); it contains *cis*-acting elements for the initiation of minus-strand RNA synthesis (Cheng & Tsai, 1999; Huang *et al*., 2001; Cheng *et al*., 2002), viral RNA long-distance movement (Chen *et al*., 2003), and the polyadenylation of plus-strand RNA (Chen *et al*., 2005). Furthermore, the host factor chloroplast phosphoglycerate kinase has been reported to interact with the 3′ UTR, and is essential for efficient BaMV accumulation in plants (Lin *et al*., 2007). A few differentially expressed genes from *N. benthamiana* plants infected with BaMV were recently identified in viral infection cycles (Cheng *et al*., 2010). One of these cDNA fragments had similarities to a GST from *N. tabacum* (Cheng *et al*., 2010).

This study is involved in a cloning of the full-length new tau group GTS gene *NbGSTU4* from the *N. benthamiana* plant. NbGSTU4 not only binds to the 3′ UTR of the BaMV RNA, but also enhances the viral RNA replication *in vitro*. This study details the role of the NbGSTU4 in BaMV infection cycle.

## Materials and Methods

### Plasmid construction

An ACCA3 cDNA fragment (200 bp) screened from cDNA-AFLP (Cheng *et al*., 2010) showing a 94% sequence identity to C-7 mRNA sequence of *N. tabacum* (accession number X64399 in NCBI database) was cloned into pTRV2 using *EcoR*I site to generate pTRV2/ACCA3 for virus-induced gene silencing (VIGS). The primer set designed for cloning the coding region of the ortholog gene from *N. benthamiana* is based on *N. tabacum* C-7 mRNA sequence. The forward primer is Nb_C-7/BamHI/F, 5′-GGATCCATGGCTGACGAAGTTGTCC-3′ (*BamH*I site is underlined); and the reverse primer is Nb_C-7/XhoI/STOP/R, 5′-GTTACTCGAGGGCAATGCCCCACTTTTG-3′ (*Xho*I site is underlined). The PCR product was cloned into pGEM-T easy vector and sequenced. The restriction sites *BamH*I/*Sal*I and BamHI/*Xho*I flanking the coding region were used for cloning to pBImGFP and pGEX-41 for plant and *E. coli* expression, respectively. For pNbGSTU4/S13A construction, the mutant fragment was amplified from pNbGSTU4 using BamHI-NbGSTU4+1, 5′-GGATCCATGGCTGACGAAGTTGTCCT-3′, and NbGSTU4-54 mutant, 5′-CCTCATCCCAAACATGGCTACATAGGTATCCAA-3′, to synthesize a 60-bp fragment which was than used as a megaprimer for a second PCR with primer mGFP-31, 5′-GTTCTTCTCCTTTACTAGTCAGATC-3′. The amplified DNA fragment was gel purified and cloned into pGEM-T easy for sequencing. A correct recombinant clone was digested with *BamH*I and *Spe*I, and inserted into the corresponding region of pNbGSTU4. For measuring the knockdown efficiency of various *NbGSTs* in plants, we used real-time PCR with the primer sets: *NbGSTU1* (F: 5′-GATGGCAGAAGTGAAGTTG-3′ and R: 5′-GCTCCTAGCCAAAATSCCA-3′); *NbGSTF1* (F: 5′-GGCTTCAAGATTAACCTGGGA-3′ and R: 5′-GCCAARATATCAGCACACC-3′); *NbGSTU2* (F: 5′-GTAGGCATAAAACCAGCTGTAGT-3′ and R: 5′-GTGAGTACATTGAWGAAGTTTGG-3′); and *NbGSTU3* (F: 5′-GCATAAGAATAAAACCAACTAGTAA-3′ and R: 5′-GTGAGTACATTGAWGAAGTTTGG-3′).

### Virus-induced gene silencing (VIGS)

The control plasmid, pTRV2/Luc, containing a portion of Luciferase gene was constructed. Plasmids pTRV2/Luc and pTRV2/ACCA3 (the 200 bp derived from *NbGSTU4*) were transformed into the *Agrobacterium tumefaciens* C58C1 strain by electroporation. To knock down the expression level of *NbGSTU4* in *N. benthamiana*, the *A.* tumefaciens C58C1 containing pTRV1, pTRV2/Luc or pTRV2/ACCA3 was cultured to OD_600_ = 1 at 30°C before induction by the addition of 130 μM acetosyringone in 10 mM MgCl_2_ for 3 h at room temperature. The pTRV2/Luc- or pTRV2/ACCA3-containing *A. tumefaciens* C58C1 was mixed with pTRV1-containing *A. tumefaciens* C58C1 at a 1 : 1 volume ratio. The 1st and 2nd leaves were infiltrated with the mixed broth at the four-leaf stage (seedlings with two cotyledons and two leaves). Then 200 ng of BaMV, PVX, CMV or TMV virion RNA was inoculated onto the 6th leaf when it was mature. Total RNAs and proteins were extracted from the leaves at 3 dpi and measured for the mRNA and viral coat protein levels, respectively. For the protoplast inoculation assay, protoplasts prepared from the 6th leaf were transfected with 1 μg of BaMV virion RNA. The levels of *NbGSTU4* mRNA and viral coat proteins were measured at 24 h post-inoculation.

### Protoplast inoculation and viral RNA quantification

Four grams of sliced *N. benthamiana* leaves were digested with 25 ml of enzyme solution (0.1% bovine serum albumin, 0.6 mg ml^−1^ pectinase and 12 mg ml^−1^ cellulase in 0.55 M Mannitol/2-(*N*-morpholino)ethanesulphonic acid pH 5.7) at 25°C overnight. The mesophyll cells were isolated and transfected with 2 μg of virion RNA (*c*. 4 × 10^5^ cells for each sample) with the help of polyethyleneglycol. Finally, the transfected protoplasts were incubated under constant light (15 μmol m^−2^ s^−1^) at 25°C for 24 or 48 h. For Northern blotting analysis, the total RNA was extracted from protoplasts (2.5 × 10^5^ cells), glyoxalated, electrophoresed through a 1% agarose gel and transferred to a Zeta-Probe membrane (Bio-Rad), as described previously (Tsai *et al*., 1999). The hybridization probe, a 0.6-kb ^32^P-labelled RNA transcript derived from the *Hind*III-linearized pBaMV-O/SB2.6 (Huang & Tsai, 1998), is complementary to the 3′-end of positive-strand BaMV RNA. The banding signals were scanned and quantified using a PhosphorImager (BAS-2500; Fujifilm, Tokyo, Japan).

The qRT-PCR technique was used to detect both BaMV plus- and minus-strand genomic RNAs. The cDNA synthesis reaction was performed according to the manufacturer's instructions using ImProm-II™ Reverse Transcriptase (Promega) with the primers Oligo dT(25T), and BaMV+1 (5′-GAAAACCACTCCAAACGAAA-3′) for the plus- and minus-strand, respectively. qPCR for quantifying the accumulation of BaMV genomic RNA and minus-strand RNA, primers BaMV+1766 (5′-CACATCCGGCACTTACCA-3′) and BaMV-2002 (5′-ATGTATCACGGAAATAAGAGTT-3′) were used in the reaction containing a 1000× dilution of SYBR green I (Cambrex Bio Science Rockland Inc., Rockland, ME, USA). qPCR was performed in 0.2-ml PCR tubes with 0.6 mM primer, 0.2 mM of each deoxynucleoside triphosphate, 10 mM Tris-HCl pH 8.8, 1.5 mM MgCl_2_, 50 mM KCl, 0.1% Triton X-100, 2 μl of cDNA, 3 units of Taq DNA polymerase (Promega) and RNase-free water to a final volume of 20 μl. Cycling conditions began with an initial hold at 95°C for 5 min, followed by 30 cycles of 94°C for 30 s, 56°C for 30 s and 72°C for 30 s. Reactions were carried out in a RotorGene 3000 (Corbett Research, Sydney, Australia) with data acquisition at 72°C on the channel with excitation at 470 nm and detection at 585 nm using a high-pass filter for both plus- and minus-strand. All samples were run at least twice, and the reaction without template or reverse transcriptase was performed as a negative control, and *actin* gene were taken for normalization.

### UV-crosslinking

The purified proteins (GST and GST-NbGSTU4) after dialysis against the buffer containing 20 mM Tris-HCl pH 7.6, 20 mM NaCl, 2 mM DTT, 5% glycerol were incubated with labeled r138/40A (the 3′ UTR of BaMV) or Ba-77 (the 3′-end of BaMV minus-strand) individually for 20 min on ice in a binding buffer (20 mM Tris-HCl pH 7.6, 2 mM MgCl_2_, 50 mM KCl, 2 mM DTT, 5% glycerol), and irradiated with a 254-nm-wavelength UV lamp (Stratagene, LaJolla, CA, USA, UV stratalinkerTM 1800) on ice for 10 min. After irradiation, the samples were treated with 40 μg of RNase A for 30 min at 37°C, boiled in 1X SDS sample buffer, and electrophoresed on a 12% or 14% sodium dodecylsulphate polyacrylamide gel. GSH was added at a different concentration (50, or 100 mM) in the UV-crosslinking reaction.

### *In vitro* RdRp assay

The replication complex of BaMV used for an *in vitro* RdRp assay was purified from infected *N. benthamiana* as described previously (Cheng *et al*., 2001; Lin *et al*., 2005). To analyze the exogenous template activity, the replication complex of BaMV was 1.5% NP40-solublize- and micrococcal nuclease-treated. The RdRp assay was carried out in a 50-μl reaction containing 5 μl of 1.5% NP40-solublized RdRp fraction, 2 mM each of ATP, CTP, and GTP, 2 μM UTP, 0.066 μM [α-^32^P]UTP (3000 Ci mmol^−1^, Dupont-NEN), 4.8 mg ml^−1^ of bentonite, 10 mM dithiothreitol, 3 mM MgCl_2_, and 50 ng of RNA template and incubated at 30°C water bath for 1 h. The RNA products were extracted with phenol-chloroform and precipitated with ethanol. For test the effects of different redox agents, 50 mM of GSH, GSSG, DTT, and H_2_O_2_ were added in the reaction. Each reagent was dissolved in 50 mM Tris buffer and adjusted pH value to 7.0 before used.

## Results

### *NbGSTU4* is required for efficient BaMV replication

**Fig. 1:**
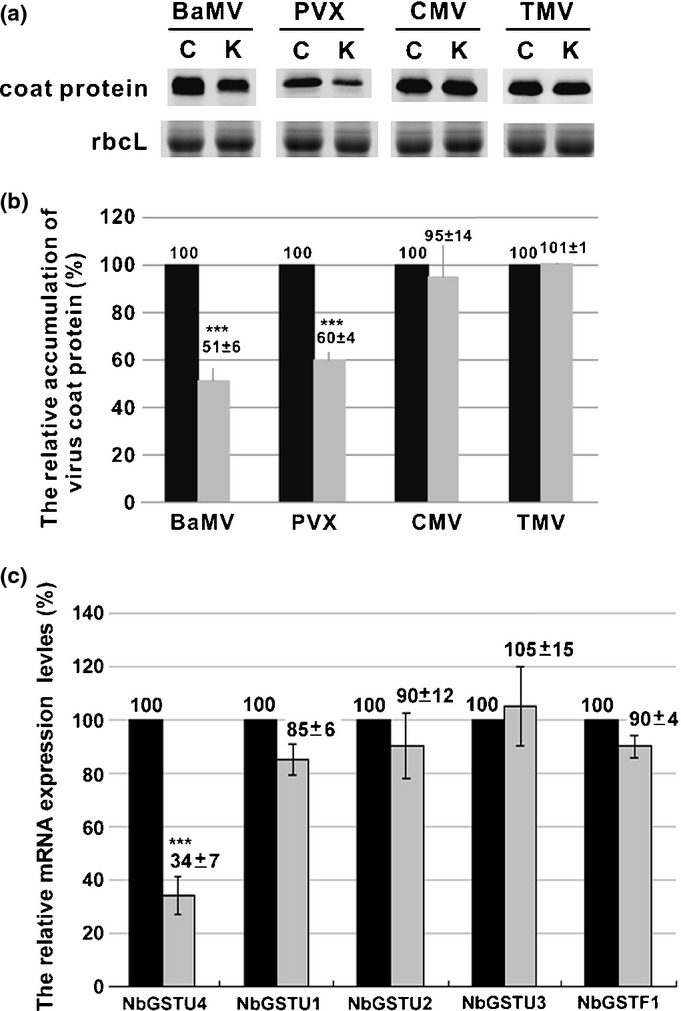
The characterization of *NbGSTU4*-knockdown *Nicotiana benthamiana* plants. (a) Western blot analysis of *Bamboo mosaic virus* (BaMV), *Potato virus X* (PVX), *Cucumber mosaic virus* (CMV) and *Tobacco mosaic virus* (TMV) coat protein in *NbGSTU4*-knockdown (indicated as K) and control (*Luc*-knockdown indicated as C) *N. benthamiana* plants 3 d post inoculation. Total proteins were extracted from inoculated leaf, separated on a 12% SDS-polyacrylamide gel, and probed with anti-coat protein antiserum. rbcL indicates the large subunit of RuBisCo stained with Coomassie Blue as a loading control. (b) A histogram of coat protein accumulation derived from (a). (c) The accumulation levels of different types of GST mRNA indicated in *NbGSTU4*-knockdown and control (*Luc*-knockdown) plants were measured by real-time RT-PCR. Control plants, black bars; NbGSTU4 knockdown plant, grey bars. The numbers shown above the statistic bar were the average ± SE of at least three independent experiments. Asterisks indicate statistically significant differences between the indicated group analyzed by Student's *t*-test (***, *P* < 0.001).

The fragment of cDNA of a GST homolog upregulated in *N. benthamiana* following BaMV infection was identified by using the cDNA-AFLP technique in our previous study (Cheng *et al*., 2010). An experiment using the *Tobacco rattle virus* (TRV)-based virus-induced gene silencing (VIGS) technique (Lin *et al*., 2007) to knock down the expression of this gene (designated hereafter as *NbGSTU4*) in *N. benthamiana* showed that the accumulation levels of the BaMV coat protein in the knockdown plants was reduced to 51% of that of the control plants. A similar reduction level was also found in another member of the *Potexvirus*, *Potato virus X* (PVX) (Fig. 1a,b). However, no significant interference occurred in the accumulation of *Cucumber mosaic virus* (CMV) and *Tobacco mosaic virus* (TMV) when inoculated into the knockdown plants (Fig. 1a,b). The knockdown efficiency of this gene was also measured by quantitative real-time PCR, and was shown to be *c*. 34% of that of the control plants (Fig. 1c). These results suggest that this GST-homolog is involved in the infection cycle of the *Potexvirus*. To investigate further, the full-length coding sequence of this *N. benthamiana* GST homolog was amplified by RT-PCR using the primers designed according to the *C-7* cDNA sequence from *N. tabacum*. Four clones were sequenced, and they had the same sequence of the gene that was isolated from *N. benthamiana* (accession number JF915552). The amino acid sequence of this cDNA has 87% identity to the GST C-7 of *N. tabacum* (Takahashi & Nagata, 1992) (Supporting Information Fig. S1). Because C-7 of *N. tabacum* has been grouped into the tau GST family (Edwards *et al*., 2000), and the nucleotide sequence of this cDNA clone shows an 80% identity to that of *NbGSTU2* in *N. benthamiana* (Dean *et al*., 2005), this study consequently designated this gene as *NbGSTU4* (the fourth tau GST gene identified from *N. benthamiana*). To examine whether knocking down the expression of *NbGSTU4* by using VIGS would also reduce the expression of other homologous genes in *N. benthamiana*, an inspection, using real-time PCR with specific primers, of the mRNA levels of *NbGSTU1*, *NbGSTU2*, *NbGSTU3* and *NbGSTF1*in the *NbGSTU4*-knockdown plants was required. The results indicated that the expression levels of the other homologous genes tested in the *NbGSTU4*-knockdown plants were insignificant unlike those of the control plants (Fig. 1c). The results suggest that the knockdown expression of *NbGSTU4* is specific, and the consequence of this knockdown in the *N. benthamiana* plant is mainly derived from the expression reduction of *NbGSTU4* (a 66% reduction, Fig. 1c). Furthermore, we could not locate any significant morphological changes in the knockdown plants in size, shape, and height compared to the control plants (inoculated with a TRV vector-carrying luciferase gene) (Fig. S2).

**Fig. 2:**
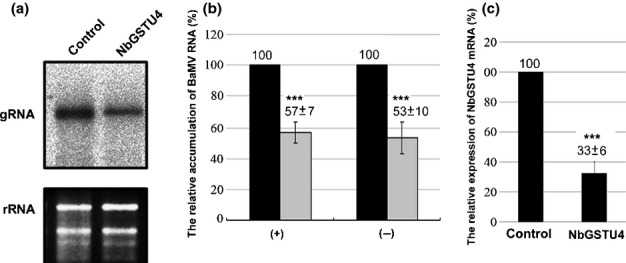
The accumulation levels of *Bamboo mosaic virus* (BaMV) RNA in *NbGSTU4*-knockdown protoplasts (a) Northern blot analysis of BaMV RNA in *NbGSTU4*-knockdown and control (Luc-knockdown) *N. benthamiana* protoplasts inoculated with BaMV RNA 48 h post-inoculation. rRNA indicates the loading control. (b) The accumulation levels of the plus- and minus-strand BaMV RNA in *NbGSTU4*-knockdown *N. benthamiana* protoplasts was measured by real-time RT-PCR. (c) The expression levels of *NbGSTU4* in control and *NbGSTU4*-knockdown *N. benthamiana* protoplasts were measured by real-time RT-PCR. Control plants, black bars; NbGSTU4, grey bars. The numbers shown above the statistic bar were the average ± SE of at least three independent experiments. Asterisks indicate statistically significant differences between the indicated group analyzed by Student's *t*-test (***, *P* < 0.001).

In order to clarify which step of the BaMV infection cycle was affected by reducing the *NbGSTU4* expression, a protoplast assay was performed to exclude cell-to-cell movement. Northern blot analysis of RNA samples, derived from *NbGSTU4*-knockdown protoplasts transfected with the BaMV for 48 h, showed that the accumulation level of the BaMV genomic RNA was reduced compared to that of the control protoplasts (Fig. 2a). To quantify the precise accumulation levels of both the BaMV RNA and *NbGSTU4* mRNA in the protoplasts, real-time PCR was performed to evaluate both the viral RNA accumulation and the mRNA knockdown efficiency, respectively. The accumulation levels of both the plus- and minus-strand BaMV RNA in the knockdown protoplasts were 64% and 58%, respectively, compared to those in the control protoplasts (Fig. 2b); the ratio of the reduction in the plus- and minus-strand BaMV RNA remained the same. The expression level of *NbGSTU4* in the knockdown protoplasts was *c*. 32% of that of the control protoplasts (Fig. 2c). These results suggest that *NbGSTU4* is involved in BaMV viral RNA replication, rather than in the virus movement.

### NbGSTU4 interacts with the 3′ UTR of BaMV RNA in a glutathione dose-dependent fashion

**Fig. 3:**
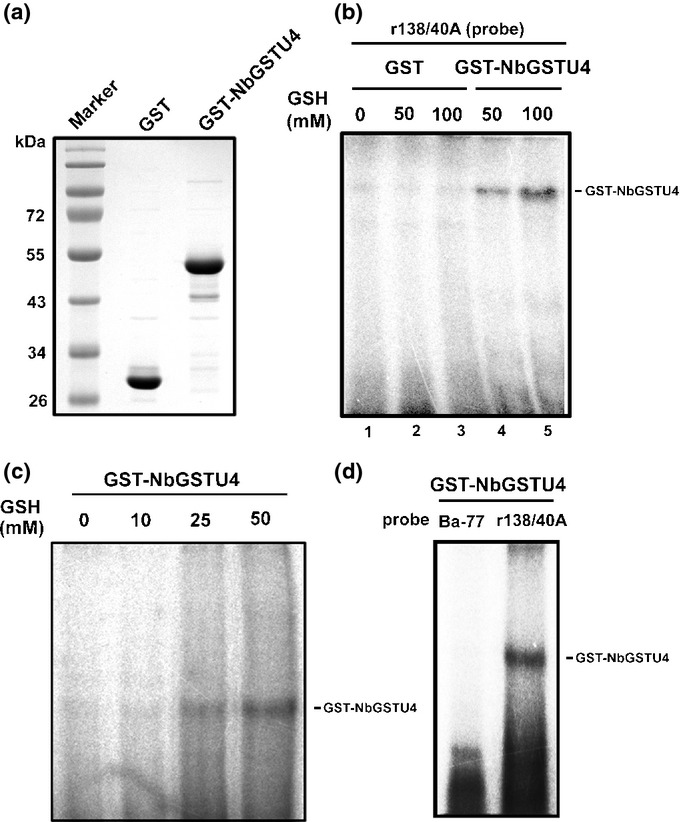
UV-crosslink of the *E. coli*-expressed GST and GST-NbGSTU4 with *Bamboo mosaic virus* (BaMV) RNAs. (a) Recombinant glutathione S-transferase (GST) and GST-NbGSTU4 proteins were expressed and purified from *E. coli* BL21, and resolved in a 12% SDS-polyacrylamide gel. (b) GST and GST-NbGSTU4 were UV-cross linked with r138/40A (178 nts, the 3′UTR containing the promoter for minus-strand RNA synthesis) in the presence of different concentration of glutathione (GSH) indicted on the top of each lane and resolved in a 14% SDS-polyacrylamide gel. The lane number is indicated at the bottom of each lane. (c) GST-NbGSTU4 was UV-cross linked with r138/40A in the presence of different concentrations of GSH (indicted at the top of each lane) and resolved in a 12% SDS-polyacrylamide gel. (d) GST-NbGSTU4 was incubated with BaMV r138/40A and Ba-77 (77 nts, the promoter for plus-strand RNA synthesis). The radioactive UV-crosslinked GST-NbGSTU4 protein is indicated.

The accumulation levels of both the plus- and minus-strands of BaMV RNA were reduced by *c*. 40% in the NbGSTU4-knockdown protoplasts (Fig. 2), suggesting that the synthesis of both strands was equally affected. One possible explanation for this is that an effect on minus-strand RNA synthesis could cause a similar reduction in plus-strand accumulation if the minus-strand RNA accumulated can produce only a fixed amount of plus-strand RNA (Chen *et al*., 2005; Wang & Nagy, 2008). We then tested whether NbGSTU4 could interact with the 3′ UTR of the BaMV genomic RNA and possibly regulate minus-strand RNA synthesis. The results derived from the UV-crosslinking assay showed that the recombinant fusion protein GST-NbGSTU4 expressed in *E. coli* (Fig. 3a) binds the 3′ UTR of BaMV (r138/40A), but no RNA binding with the fusion partner (GST) alone was observed (Fig. 3b, Lane 1).

Because GSTs bind GSH as a co-substrate/cofactor to form a functional homo or heterodimer (Dixon *et al*., 2002), GSH was added in the UV-crosslinking reactions to determine whether the interaction of GST with GSH could affect the binding. This showed that GSH could enhance the binding of NbGSTU4 with the 3′ UTR of BaMV in a higher dose concentration (50 and 100 mM; Fig. 3b, Lanes 4 and 5). Because the intracellular concentration of GSH is highly variable, ranging from *c*. 1 to as much as 10–20 mM (Meister & Anderson, 1983), we then tested whether the NbGSTU4 still can interact with the BaMV RNA at that concentration. The results shown in a dose-dependent manner indicated that the interaction could be observed in the presence of 10 mM GSH (Fig. 3c). We further tested whether NbGSTU4 can bind the 3′-terminal region of the minus-strand BaMV (Ba-77); the promoter for plus-strand RNA synthesis (Fig. 3d). The results indicated that the binding was specific to the 3′ UTR of BaMV.

### GSH enhances replication of the BaMV at an early stage

**Fig. 4:**
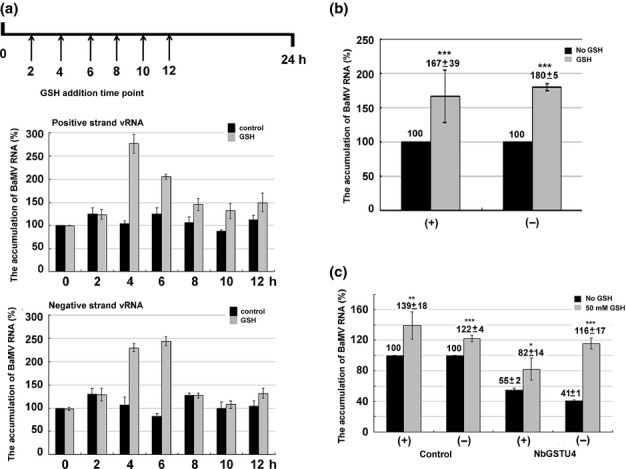
Real-time RT-PCR analysis of the accumulation of *Bamboo mosaic virus* (BaMV) RNA in the presence of glutathione (GSH) in protoplasts and plants. (a) Protoplasts were treated with GSH or Tris buffer (control) at different time points (h) post-BaMV RNA inoculation (hpi) indicated with arrows. Total RNAs were extracted at 24 hpi for real-time RT-PCR analysis. The accumulation levels of BaMV plus- and minus-strand RNA (Table S1) were indicated in the histogram. (b) The accumulation levels of BaMV plus-strand (+) and minus-strand (−) RNA in *N. benthamiana* which were co-infiltrated with the infectious BaMV cDNA clone pKB (driven by ^35^S promoter) and either Tris buffer as a control or with GSH (50mM). (c) The accumulation levels of BaMV plus-strand (+) and minus-strand (−) RNA in *NbGSTU4*-knockdown or control *N. benthamiana* plants which were co-infiltrated with pKB and either Tris buffer or GSH (50 mM) at 24 hpi. The numbers shown above the statistic bar were the averages ± SE of three independent experiments. Asterisks indicate statistically significant differences between the indicated group analyzed by Student's *t*-test (**, *P* < 0.01; ***, *P* < 0.001).

Because GSH could enhance the binding of NbGSTU4 with the 3′ UTR of BaMV RNA, a test was conducted to determine whether a simple addition of GSH into BaMV-transfected protoplasts could enhance viral accumulation. The experiment was performed by transfecting BaMV RNA into *N. benthamiana* protoplasts and adding GSH into the medium at different time points post-inoculation. The accumulation levels of the BaMV plus- and minus-strand RNA of all of the treatments were examined by real-time RT-PCR at 24 h post-inoculation (hpi). The addition of GSH at 4 and 6 hpi caused a significant enhancement in the accumulation of both BaMV plus- and minus-strand RNA (Fig. 4a). Furthermore, the *N. benthamiana* plant infiltrated directly by GSH could also significantly enhance the accumulation of both the plus- and minus-strands of BaMV RNA at 24 hpi (Fig. 4b).

The observations that GSH could enhance the NbGSTU4 binding to the BaMV 3′ UTR and that the addition of GSH stimulated BaMV accumulation suggested that the BaMV 3′ UTR binds NbGSTU4 to recruit GSH for BaMV RNA production. One of the possible mechanisms is that GSH is required for minus-strand RNA synthesis and, thus, is achieved through its interaction with NbGSTU4. If this hypothesis is correct, the accumulation levels of the BaMV in the *NbGSTU4*-knockdown plants can be restored when GSH is provided. The results validated the proposed hypothesis that providing sufficient GSH can raise the accumulation of the BaMV RNA in *NbGSTU4*-knockdown plants more than that in the control plants, especially for minus-strand RNA (Fig. 4c).

**Fig. 5:**
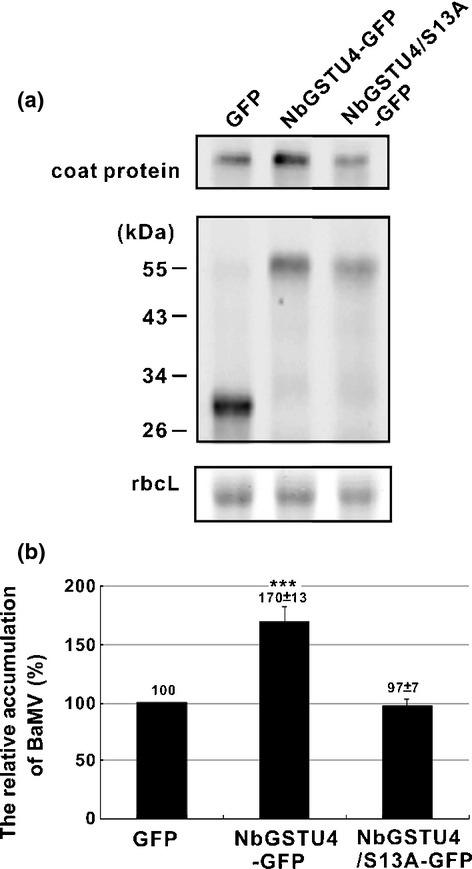
Western blotting analysis of the accumulation of *Bamboo mosaic virus* (BaMV) coat protein in NbGSTU4-GFP or its mutant expressed *N. benthamiana* plants. (a) The plants were inoculated with BaMV virion and followed by transiently expressed GFP, NbGSTU4-GFP or NbGSTU4/S13A-GFP at 2 d post-inoculation (dpi). The total proteins were extracted at 5 dpi. The protein accumulation levels were measured by western blotting with the antibody against BaMV coat protein or GFP. (b) The accumulation levels of BaMV coat protein were shown as the histogram indicated. The numbers shown above the statistic bar were the average ± SE of at least three independent experiments. Asterisks indicate statistically significant differences between the indicated group analyzed by Student's *t*-test (***, *P* < 0.001).

The hypothesis that the NbGSTU4 binding the 3′ UTR of BaMV delivers GSH to the replication complex for viral RNA synthesis was tested further by creating a mutant NbGSTU4/S13A-GFP, which abolished the GSH-binding activity by substituting the conserved GSH-binding site serine to alanine (Zeng *et al*., 2005). We then transiently expressed NbGSTU4-GFP or NbGSTU4/S13A-GFP in BaMV-inoculated *N. benthamiana* leaves. The results indicated that the accumulation of the BaMV coat protein in the NbGSTU4-GFP-expressed plant is enhanced to 170% of those of the control plants (transiently expressed GFP only) (Fig. 5). However, in NbGSTU4/S13A-GFP-expressed plants the accumulation of the BaMV coat protein was similar to that of control plants. The results confirm that the GSH-binding activity of NbGSTU4 is important for BaMV accumulation.

### GSH stimulates minus-strand RNA synthesis in an *in vitro* RdRp assay

**Fig. 6:**
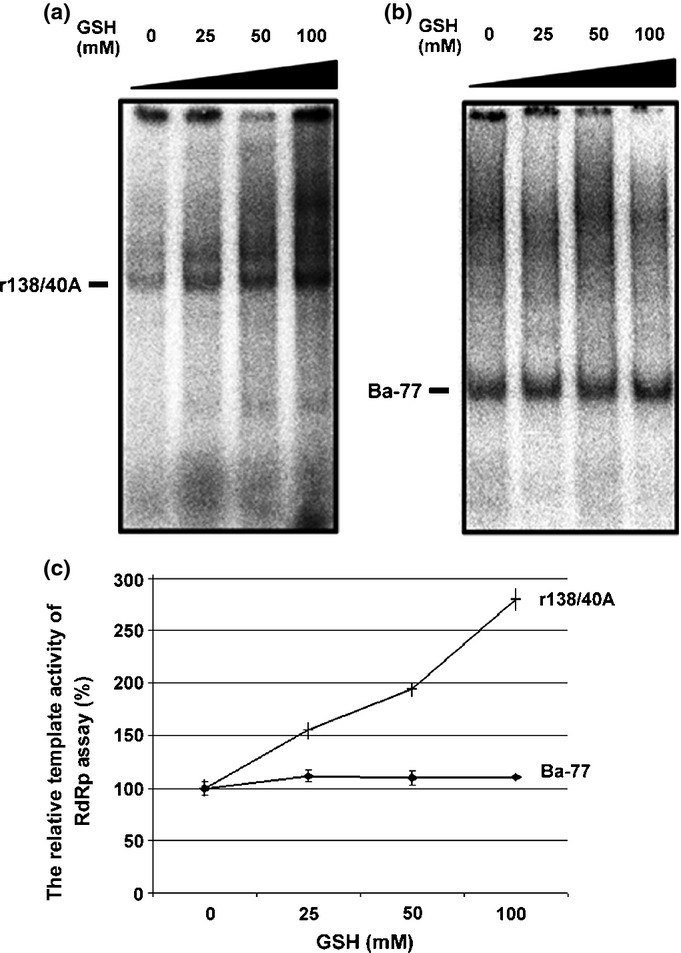
*In vitro* RdRp assay with exogenous RNA templates. Approximately 50 ng of RNA template r138/40A (the 3′ UTR of *Bamboo mosaic virus* (BaMV)) in (a) and Ba-77 (the 3′-end 77 nts of BaMV minus-strand) in (b) were incubated with BaMV RdRp complex for the *in vitro* RNA synthesis in the presence of different concentrations (0–100 mM) of glutathione (GSH) as indicated. The RdRp products labeled with [α-^32^P] UTP as indicated bands were separated on a 5% acrylamide gel and quantified by a phosphorimager. (c) The relative RdRp template activities were plotted according to the data derived from (a) and (b). The banding density of the *in vitro* RdRp assay with either r138/40A or Ba-77 was set as 100% in the absence of GSH (0 mM). Each spot on the plot was the average ± SE of at least three independent experiments.

**Fig. 7:**
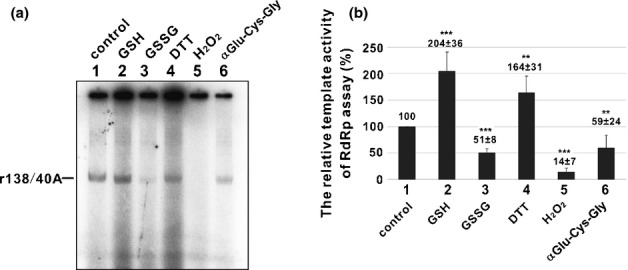
The effects of different redox agents in the *in vitro* RdRp assay. (a) Approximately 50 ng of RNA template r138/40A was incubated with *Bamboo mosaic virus* (BaMV) RdRp complex for *in vitro* RNA synthesis in the presence of 50 mM of different redox agents as indicated. (b) The relative RdRp template activities were plotted according to the data derived from (a). The banding density of the *in vitro* RdRp assay in the presence of 50 mM Tris buffer (pH 7.0) was set as 100%. The numbers shown above the statistic bar were the average ± SE of three independent experiments. Asterisks indicate statistically significant differences between the indicated group analyzed by Student's *t*-test (**, *P* < 0.01; ***, *P* < 0.001).

This study showed that NbGSTU4 and GSH can facilitate the replication of the BaMV in *N. benthamiana*. The effect of the GSH on the replication of the BaMV is suggested to be associated with minus-strand RNA synthesis. Further validation of this hypothesis required performing an *in vitro* RdRp assay with exogenous RNA templates in different concentrations of GSH (Fig. 6). The results indicate that GSH can only facilitate minus-strand RNA synthesis (using 3′ UTR of BaMV RNA, r138/40A) in a dose-dependent manner. In 100 mM GSH, r138/40A template activity could reach 2.7-fold of that without the addition of GSH in the RdRp preparation. By contrast, we could not observe the enhancement when GSH was added to plus-strand RNA synthesis (using the 3′-terminal promoter sequence of minus-strand RNA, Ba-77). We then tested whether the BaMV minus-strand RNA synthesis requires GSH specifically, or simply requires reduced conditions in an *in vitro* RdRp assay. The results indicated that the RNA synthesis rate could be enhanced significantly when reducing agents, such as GSH and DTT, are added to the reaction, whereas oxidizing agents, such as GSSG and H_2_O_2_, would reduce RNA synthesis (Fig. 7). We also tested whether the synthetic tripeptide, α-glutamate-cystein-glycine, mimicking the GSH structure, but without the γ-glutamate linkage, can have the same effect in the RdRp assay. The results indicated that the synthesized tripeptide reduced the RdRp activity (Fig. 7, Lane 6) and suggested the specificity of the GSH in BaMV RNA synthesis.

Overall, the results suggest that minus-strand RNA synthesis of the BaMV required a more reduced environment for efficient RNA synthesis. To create this condition for RNA synthesis, the viral RNA interacts with a GST, NbGSTU4, to draw the GSH into the replication complex.

## Discussion

Host factors associated with the 3′ UTR of positive-sense RNA viruses may have contradictory roles in regulating viral infection cycles. Overexpression of the 3′ UTR binding protein Nsr1p/nucleolin in *Nicotiana benthamiana* reduced the accumulation of tombusvirus RNA, and the addition of purified Nsr1p in the replicase complex inhibited the *in vitro* activity (Jiang *et al*., 2010). Knocking down the expression level of glyceraldehyde 3-phosphate dehydrogenase (GAPDH) in *N. benthamiana* could enhance accumulation of the BaMV, and the addition of recombinant GAPDH in the RdRp assay could inhibit minus-strand RNA synthesis, suggesting a role in negatively regulating BaMV infection (Prasanth *et al*., 2011). NF90 interacting with the stable 3′-terminal stem-loop structure of the dengue RNA could positively regulate its replication. A depletion of NF90 in cells could significantly diminish the production of infectious dengue viruses (Gomila *et al*., 2011). The translation elongation factor, eEF1A, not only binds to the 3′-end of the *Tomato bushy stunt virus* (TBSV) RNA, but also binds to the TBSV p33 replication protein and acts as a co-factor assisting in viral RNA replication (Li *et al*., 2009). In this study, we also found that a host factor, a GST, could interact with the 3′ UTR of BaMV RNA and positively regulate minus-strand RNA synthesis.

Plant GSTs are a group of enzymes playing a major role in protecting plants from various stresses, such as pathogen attacks (Mauch & Dudler, 1993; Li *et al*., 1997). The three known tau-class GSTs identified in *N. benthamiana* (*NbGSTU1*, *-U2* and *-U3*) have been reported to be involved in fungal infections (Dean *et al*., 2005). The new member of the 4th tau GST gene, *NbGSTU4*, identified in this study is required for BaMV efficient replication. This study provides evidence that, by using the TRV-based VIGS technique to knock down the expression of *NbGSTU4*, no effect on the expression levels of other homologous genes (the other known tau GSTs), was specific in *N. benthamiana* (Fig. 1). A survey in the rice genome identified 79 GSTs including 52 tau GSTs (Jain *et al*., 2010), among which OsGSTU4 was demonstrated to play a role in herbicide resistance (Jo *et al*., 2011). Although none of the tau GST gene in bamboo (the natural host of BaMV) has been identified yet, we believe that bamboo belonging to Poaceae family should contain as many as tau GSTs as those in rice.

Three lines of evidence help link NbGSTU4 to minus-strand RNA synthesis: (1) the *E. coli*-expressed NbGSTU4 binds to the 3′ UTR, although not to the 3′-end of the minus-strand BaMV RNA (Fig. 3); (2) the reduction or enhancement of the plus- and minus-strand RNA accumulation in knockdown or transiently expressed plants, respectively, remains the same (Fig. 2); and (3) the effect of the GSH on BaMV accumulation in protoplasts occurs only at an early time point (Fig. 4). These results suggest that minus-strand RNA synthesis requires GSH. This requirement is evident from the *in vitro* RdRp assay, which shows that the template activity of the 3′ UTR exogenous RNA can be enhanced by the addition of GSH in a dose-dependent manner, whereas the promoter for plus-strand RNA synthesis cannot be enhanced in the presence of GSH (Fig. 6).

GSTs and GSH are involved in antioxidation processes in cells. The oxidative stress (a plant anti-pathogen infection strategy) induced by virus infections (Fodor *et al*., 2001; Mathew *et al*., 2010) can be a hindrance for viral replication. Therefore, reducing the oxidative stress by providing GSH can be a crucial step for viral RNA replication in cells after infection. A possible role of *NbGSTU4* upregulated by virus infection is to provide an antioxidation environment for virus replication. The results derived from the *in vitro* RdRp assay indicate that providing GSH or DTT can enhance minus-strand RNA synthesis, but not plus-strand RNA synthesis. The likely role of NbGSTU4 is to bring GSH to the replication site and provide anti-oxidative conditions for minus-strand RNA synthesis. Because the concentration of GSH in cells is *c*. 1–20 mM, it might not provide a sufficiently strong reduced environment for viral RNA replication. The interaction of viral RNA with GST (BaMV RNA with NbGSTU4, Fig. 3) to recruit GSH and to increase the reductive condition at the viral replication site might be a good strategy.

### Conclusion

This is the first study to examine the involvement of a new plant tau-class GST protein, NbGSTU4, in BaMV viral RNA minus-strand RNA synthesis. Through the binding of NbGSTU4 to the 3′ UTR of the genomic RNA, either by providing an antioxidative condition or by changing the redox status of the replicase complex, the minus-strand RNA can be synthesized efficiently. The discovery of a plant GST protein involving the replication of a plant virus could help us to gain insights into the relationship between viral RNA replication and the GST metabolic pathway.

## Supporting Information

**Fig. S1** Amino acid sequence alignment of *Nicotiana tabacum* C-7 (accession number Q40480), and *N. benthamiana* NbGSTU4 (accession number JF915552).

**Fig. S2** The phenotype of *NbGSTU4*-knockdown *Nicotiana benthamiana* plants.

**Table S1** The relative accumulation levels of BaMV plus- and minus-strand RNA in the control (Tris buffer) and GSH (GSH treated) protoplasts
