# Supplementary material for: The glutathione transferase of Nicotiana benthamiana NbGSTU4 plays a role in regulating the early replication of Bamboo mosaic virus
Source: New Phytol. 2013 May 23;199(3):749–57. doi: 10.1111/nph.12304 (PMC3744755; doi:10.1111/nph.12304)
Supplement: Supplementary file 1 [file nph0199-0749-SD1.pdf]

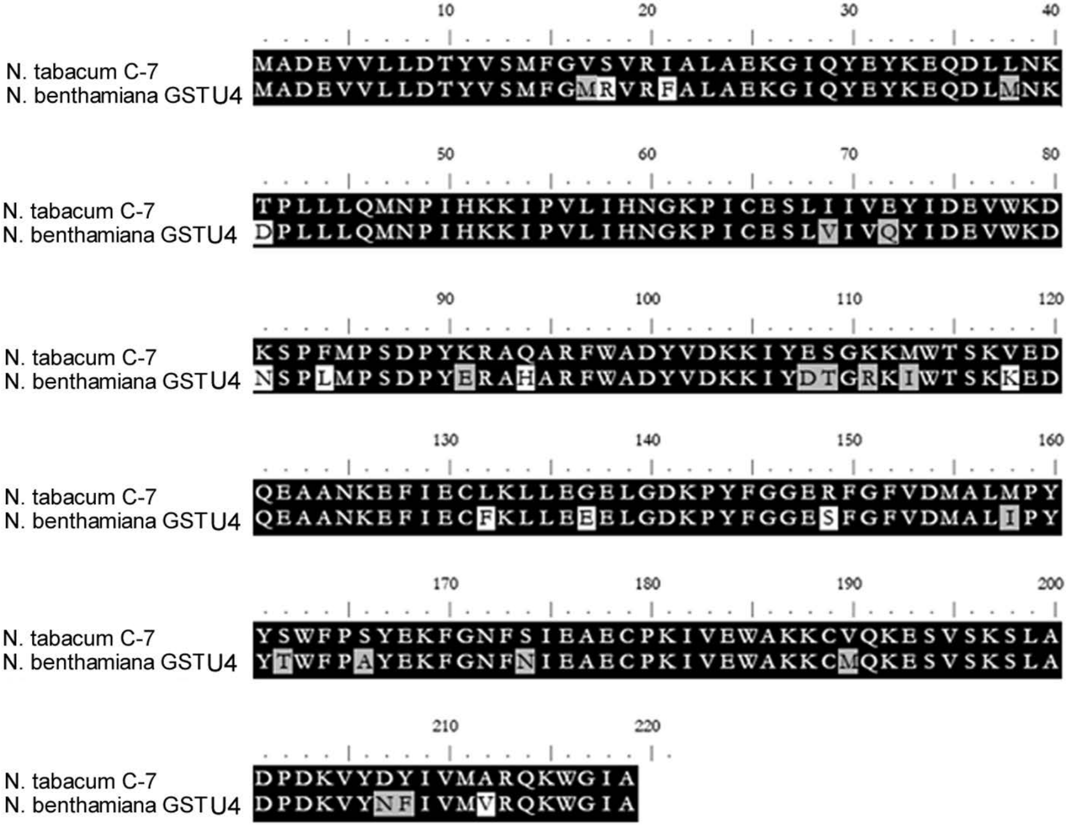


**Supplemental Figure S1.**

Amino acid sequence alignment of *Nicotiana tabacum* C-7 (accession number Q40480), and *N. benthamiana* NbGSTU4 (accession number JF915552).

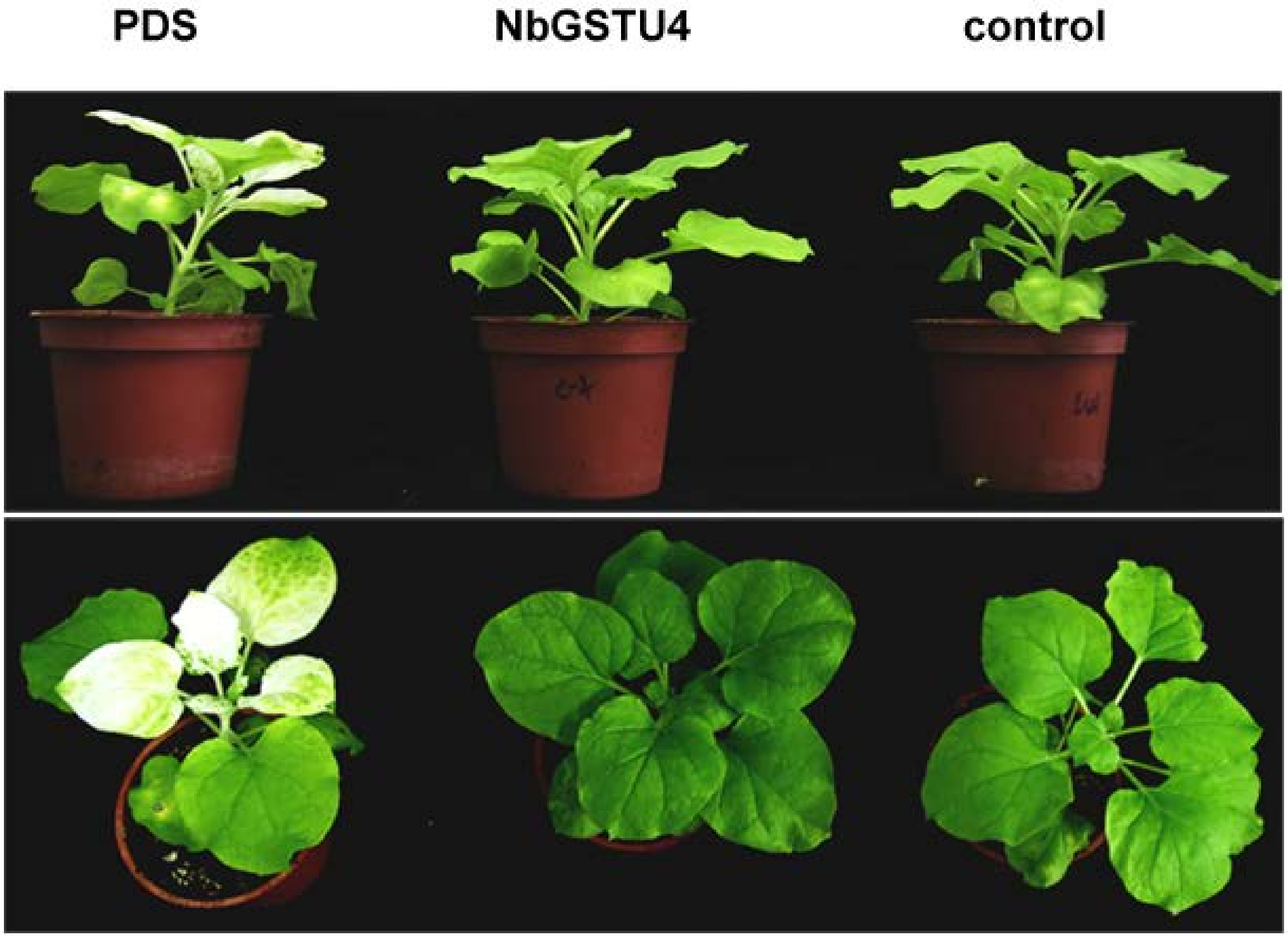


**Supplemental Figure S2.**

**The phenotype of *NbGSTU4*-knockdown *Nicotiana benthamiana* plants.**

*Agrobacterium* C58C1 containing plasmid, pTRV2/NbGSTU4 (NbGSTU4), pTRV2/phytoene desaturase (PDS), or pTRV2/luciferase (control) was co-infiltrated with *Agrobacterium* containing pTRV1 into 4-week-old *N. benthamiana*. The photo was taken at 10 days of post infiltration on the plants indicated. *PDS*-knockdown plant showed a photo-bleaching phenotype, but no obvious difference of the phenotype between *NbGSTU4*-knockdown and control plants.

| | GSH addition time point | 0 hr | 2 hr | 4 hr | 6 hr | 8 hr | 10 hr | 12 hr |
|---|---|---|---|---|---|---|---|---|
| positive strand | control | 100 | 124±14 | 104±7 | 125±14 | 106±13 | 88±3 | 113±10 |
| | GSH treatment | 100±1 | 124±10 | 277±20 | 205±6 | 145±13 | 132±16 | 150±20 |
| negative strand | control | 100 | 130±12 | 107±17 | 82±6 | 128±4 | 100±13 | 104±13 |
| | GSH treatment | 98±4 | 129±14 | 230±9 | 244±10 | 128±6 | 108±8 | 132±11 |

**Supplemental Table S1.**
The relative accumulation levels of BaMV plus- and minus-strand RNA in the control (Tris buffer) and GSH (GSH treated) protoplasts. All the data were the average and the standard error of three independent experiments. The accumulation level of the BaMV genomic RNA in the control protoplasts was set as 100 %
